# Correlation between vitamin D levels and blood pressure in elderly hypertensive patients with osteoporosis

**DOI:** 10.3389/fmed.2024.1396254

**Published:** 2024-05-21

**Authors:** Tianlong Wu, Zebin Lin, Chizhen Wang, Xia Liu

**Affiliations:** ^1^Department of Geriatrics, Zhongshan Hospital Affiliated to Xiamen University, Xiamen, Fujian, China; ^2^The First Affiliated Hospital of Bengbu Medical College, Bengbu, Anhui, China

**Keywords:** hypertension, vitamin D, osteoporosis, elderly, ambulatory blood pressure

## Abstract

**Objectives:**

The association between vitamin D and blood pressure in elderly patients with hypertension complicated by osteoporosis remains unclear. The objective of this study is to explore whether vitamin D deficiency contributes to elevated blood pressure in elderly individuals with both hypertension and osteoporosis.

**Methods:**

This study represents a single-center retrospective observational investigation carried out at the Zhongshan Hospital Affiliated to Xiamen University. Ambulatory blood pressure, bone density, vitamin D levels, and additional laboratory parameters were collected upon admission. The association between vitamin D and ambulatory blood pressure outcomes was assessed using Spearman correlation tests and partial correlation analyses. The relationship between vitamin D and changes in blood pressure was analyzed through Generalized Additive Models, and threshold analysis was conducted to explore potential thresholds.

**Results:**

139 patients with newly diagnosed osteoporosis were consecutively included (mean age 73 years, 84.9% female). There is a negative correlation between 25-(OH) D3 and 24 h mean systolic blood pressure (mSBP), diurnal mSBP, nocturnal mSBP, maximum SBP, respectively. The results of the generalized additive model analysis show that there is a nonlinear relationship between 25-(OH) D3 and 24 h mSBP, diurnal mSBP, nocturnal mSBP, respectively. After determining the critical point of 25-(OH) D3 as 42 nmol/L, a segmented linear regression model was used to calculate the effect size and 95% confidence interval on both sides of the critical point. When 25-(OH) D3 is ≤42 nmol/L, it significantly negatively correlates with 24 h, diurnal, and nocturnal mean SBP. Conversely, when 25-(OH) D3 exceeds 42 nmol/L, there is no statistically significant association with 24 h, diurnal, or nocturnal mSBP.

**Conclusion:**

There was a significant negative correlation between vitamin D levels and blood pressure levels in elderly patients with hypertension and osteoporosis.

## 1 Introduction

Hypertension is a common chronic disease and has been recognized as the most prevalent risk factor for cardiovascular disease, renal failure and stroke (1). According to Global Burden of Diseases, Injuries, and Risk Factors Study, high systolic blood pressure is the leading risk factor for death in China, accounted for 2.54 million (95% UI 2.26 million to 2.82 million) deaths in 2017 (2). There are many factors associated with high blood pressure, such as age and gender (3). In recent years, studies have suggested that vitamin D deficiency contribute to the development of hypertension, which may because the vitamin D deficiency may increase activity of the RAAS (4, 5). In addition, some studies have found that vitamin D supplementation reduces blood pressure (6–8). Another study demonstrated that vitamin D supplementation may reduce blood pressure by suppress NO signaling through lowering serum TSP-1 levels (9). This suggests that vitamin D may be a potential treatment for hypertension.

Osteoporosis is a metabolic disease in the elderly, which can cause bone mass loss, bone structure deterioration, and then lead to increased bone fragility and even fracture. Vitamin D is a fat-soluble steroid that is involved in bone metabolism by regulating calcium and phosphorus metabolism to maintain blood calcium levels and promote bone development (10). There was an association between vitamin D with bone health (11). Studies have shown that vitamin D supplementation can reduce hip or non-vertebral fractures by 20 to 30% (12–14). Therefore, vitamin D is recommended for the prevention of osteoporosis and subsequent fractures.

Since vitamin D deficiency is associated with both hypertension and osteoporosis, what is the effect of vitamin D deficiency on blood pressure in patients with hypertension and osteoporosis, especially in the elderly, who are more susceptible to both conditions, and is vitamin D supplementation a potential treatment option.

At present, the relationship between vitamin D and blood pressure has not been clarified in elderly hypertensive patients with osteoporosis. The aim of this study was to investigate whether vitamin D deficiency leads to higher blood pressure in elderly hypertensive patients with osteoporosis.

## 2 Materials and methods

### 2.1 Study population

This study represents a single-center retrospective observational investigation carried out at the Geriatrics Department of Zhongshan Hospital Affiliated to Xiamen University. Over the period from October 2022 to August 2023, a cohort of 139 elderly patients with hypertension, newly diagnosed with osteoporosis, were included in the study.

#### 2.1.1 Inclusion criteria and definitions

(1) Age ≥ 65 years; (2) Primary hypertension is diagnosed based on the criteria outlined in the Chinese Guidelines for the Prevention and Treatment of Hypertension. This involves excluding hypertension caused by other secondary factors and recording systolic blood pressure ≥ 140 mmHg and/or diastolic blood pressure ≥ 90 mmHg on three or more separate occasions, or already taking antihypertensive medication. (3) Primary osteoporosis is diagnosed according to the definition provided in the Diagnosis and Treatment Guidelines for Primary Osteoporosis. It is characterized as a systemic bone disease featuring low bone mass and increased bone fragility, with exclusion of secondary factors. This study primarily concentrates on populations with postmenopausal osteoporosis and elderly osteoporosis.

#### 2.1.2 Exclusion criteria

(1) Individuals who have taken or are currently taking any vitamin D or its derivatives preparations; (2) End-stage diseases, including heart failure (New York Heart Association class III-IV), severe renal dysfunction (CKD stage IV-V), severe hepatic dysfunction (Child C); (3) Malignant tumors, including hematological malignancies; and (4) Acute phases of diseases such as acute organ failure, stroke, trauma, etc.

This study was approved by the ethics committee of the Zhongshan Hospital Affiliated to Xiamen University, and the requirement for informed consent was waived. The investigation conformed to the principles outlined in the Declaration of Helsinki.

### 2.2 Measurement of indicators of interest

A non-invasive, portable, fully automatic monitoring device (DMS-ABP, DMS Company, Beijing) was employed for blood pressure measurements. The appropriately sized cuff was chosen based on the patient’s arm circumference and worn on the left arm. Blood pressure monitoring was conducted from 8 AM in the morning to 8 AM the following day, with measurements set at intervals of every 30 min during both day and night. Valid readings were defined as achieving 70% or more of the expected readings, with a minimum of 20 valid readings during the daytime and at least 7 valid readings during the nighttime.

The measurement of 25-(OH) D3 and other bone metabolism markers was conducted at the clinical testing center using an automated chemiluminescence immunoassay analyzer (ADVIA Centaurxp, Siemens, Germany). The results of 25-(OH) D3 testing are categorized as follows: levels below 50 nmol/L are deemed insufficient, those within the range of 50–75 nmol/L are considered deficient, and values exceeding 75 nmol/L are classified as sufficient.

### 2.3 Statistical analysis

The normality of data distribution was assessed employing the Kolmogorov–Smirnov test. Data conforming to a normal distribution were expressed as mean ± SD, while skewed datasets were described as median [25th percentile, 75th percentile]. Group comparisons for normally distributed data with homogeneity of variance were conducted using analysis of variance (ANOVA), with subsequent pairwise comparisons facilitated by the LSD method. In cases of normally distributed data with heterogeneous variance, Welch’s test was utilized for intergroup comparisons. For skewed datasets, the Kruskal-Wallis test was applied for intergroup comparisons. Spearman correlation tests and partial correlation analyses were employed to evaluate the association between 25-(OH) D3 and dynamic blood pressure results. The relationship between 25-(OH) D3 and blood pressure changes was analyzed using generalized additive models, followed by threshold analysis. Statistical significance was set at *p* < 0.05 (two-sided). Data analysis was performed using R language (R for win version 4.3.0, R Foundation for Statistical Computing, Vienna).

## 3 Results

### 3.1 Baseline characteristic

According to the previously set inclusion and exclusion criteria, 139 patients with newly diagnosed osteoporosis were consecutively included (mean age 73 years, 84.9% female). According to the 25-(OH) D3 test results, patients with ≤50 nmol/L were assigned to the deficiency group, patients with 50–75 nmol/L were assigned to the insufficient group, and patients with >75 nmol/L were assigned to the normal group. According to the difference test results between groups, there were significant differences in 24 h mSBP, diurnal mSBP, nocturnal mSBP, NBPDR DBP, maximum SBP and CRP among the three groups. Further pairwise comparison results showed that the 24 h mSBP, nocturnal mSBP, NBPDR DBP, maximum SBP and CRP of patients in the deficiency group were higher than those of patients in the normal group. Compared with the insufficient group, the nocturnal mSBP of patients in the normal group was higher. There was no significant difference in bone mineral density and bone metabolism indicators. The baseline characteristics are shown in Table 1.

**Table 1:** Baseline characteristics of subjects.

	Total (*n* = 139)	Deficiency (*n* = 38)	Insufficient (*n* = 61)	Normal (*n* = 40)	*p* value
Age (year)	73 ± 10	72 ± 11	74 ± 10	73 ± 11	0.79
Male (*n*, %)	21 (15.1)	6 (15.8)	8 (13.1)	7 (17.5)	0.83
24 h mDBP (mmHg)	77 [70, 84]	77 [71, 84]	77 [72, 85]	77 [68, 81]	0.28
24 h mSBP (mmHg)	139 ± 17	146 ± 20	139 ± 16	135 ± 15a	0.02
Diurnal mSBP (mmHg)	140 [128, 150]	145 [132, 161]	140 [128, 152]	137 [127, 146]	0.06
Diurnal mDBP (mmHg)	79 ± 11	80 ± 13	80 ± 10	76 ± 9	0.22
Nocturnal mSBP (mmHg)	136 [123, 151]	144 [131, 155]	133 [119, 151]^a^	135 [119, 141]^a^^b^	0.01
Nocturnal mDBP (mmHg)	74 [67, 83]	75 [69, 87]	75 [67, 84]	74 [63, 79]	0.07
NBPDR, SBP (%)	2.39 ± 8.05	0.72 ± 9.46	2.99 ± 7.37	3.05 ± 7.46	0.33
NBPDR, DBP (%)	4.20 ± 8.80	1.15 ± 10.67	5.28 ± 7.64a	5.68 ± 7.97a	0.04
Maximum SBP	181 [167, 201]	191 [171, 208]	185 [163, 203]	174 [159, 186]^a^	0.01
Maximum DBP	110 ± 15	112 ± 17	111 ± 15	108 ± 13	0.50
Minimum SBP	105 ± 15	109 ± 18	105 ± 13	103 ± 15	0.20
Minimum DBP	53 ± 11	54 ± 13	54 ± 11	51 ± 10	0.34
lumbar vertebra (T)	−2.4 [−3.0, −1.4]	−2.3 [−3.0, −1.3]	−2.5 [−3.0, −1.4]	−2.4 [−3.3, −1.5]	0.85
lumbar vertebra BMD	0.89 [0.82, 1.01]	0.90 [0.82, 1.04]	0.89 [0.82, 1.01]	0.90 [0.78, 1.003]	0.80
Left neck of femur (T)	−2.5 [−3.0, −2.1]	−2.5 [−2.9, −2.3]	−2.5 [−3.0, −2.0]	−2.4 [−3.0, −2.0]	0.51
Left neck of femur BMD	0.70 [0.62, 0.76]	0.68 [0.65, 0.73]	0.70 [0.62, 0.76]	0.70 [0.62, 0.76]	0.67
Right neck of femur (T)	−2.6 [−3.0, −2.2]	−2.6 [−2.9, −2.2]	−2.6 [−3.0, −2.2]	−2.5 [−3.0, −2.1]	0.98
Right neck of femur BMD	0.68 [0.62, 0.74]	0.67 [0.64, 0.74]	0.68 [0.62, 0.73]	0.70 [0.62, 0.76]	0.99
Left hip (T)	−2.0 [−2.5, −1.5]	−2.0 [−2.6, −1.6]	−1.9 [−2.5, −1.5]	−2.0 [−2.6, −1.5]	0.91
Left hip BMD	0.76 [0.69, 0.82]	0.76 [0.70, 0.82]	0.77 [0.69, 0.82]	0.75 [0.66, 0.82]	0.96
Right hip (T)	−2.0 [−2.4, −1.5]	−2.1 [−2.6, −1.4]	−2.0 [−2.3, −1.4]	−2.0 [−2.4, −1.5]	0.86
Right hip BMD	0.76 [0.69, 0.82]	0.76 [0.70, 0.83]	0.76 [0.71, 0.83]	0.76 [0.70, 0.82]	0.91
Minimum bone density (T)	−2.9 [−3.4, −2.1]	−2.9 [−3.5, −2.5]	−2.9 [−3.3, −2.5]	−2.8 [−3.4, −2.4]	0.66
WBC,10^9^/L	6.3 ± 1.4	6.4 ± 1.6	6.3 ± 1.3	6.4 ± 1.4	0.90
RBC,10^9^/L	4.2 ± 0.57	4.3 ± 0.7	4.3 ± 0.55	4.2 ± 0.47	0.35
Platelet,10^12^/L	213 ± 56	225 ± 73	213 ± 42	203 ± 54	0.31
IPTH, pg./mL	55 [39, 72]	57 [40, 83]	52 [40, 71]	60 [38, 70]	0.63
β-Crosslaps, ng/mL	0.48 [0.26, 0.7]	0.43 [0.27, 0.7]	0.52 [0.33, 0.7]	0.45 [0.18, 0.6]	0.45
Osteocalcin, ng/mL	17 [13, 21]	17 [13, 24]	17 [13, 24]	17 [11, 21]	0.78
25-(OH) D3, ng/mL	64 ± 21	39 ± 7.1	62 ± 7.1^a^	91 ± 13^a^^b^	<0.001
PINP, ng/mL	51 ± 21	56 ± 26	50 ± 17	47 ± 21	0.26
Albumin, g/L	39 ± 3.6	39 ± 3.7	40 ± 3.4	40 ± 3.4	0.51
TC, mmol/L	4.7 ± 1.2	4.9 ± 1.3	4.7 ± 1.3	4.3 ± 1.01	0.11
HDL, mmol/L	1.32 ± 0.34	1.25 ± 0.32	1.36 ± 0.33	1.34 ± 0.38	0.33
LDL, mmol/L	3.01 ± 0.91	3.2 ± 1.01	3.1 ± 0.91	2.8 ± 0.77	0.08
K+, mmol/L	3.9 ± 0.4	4 ± 0.5	3.9 ± 0.38	4 ± 0.33	0.47
Folic acid, nmol/L	30 [21, 42]	30 [18, 47]	29 [21, 39]	31 [21, 41]	1.00
CRP, mg/L	2.7 [1.2, 4.1]	3 [1.4, 5.7]	2.8 [1.4, 4.4]	1.9 [0.84, 3.4]^a^	0.05
Total bilirubin, umol/L	11 [9.4, 14]	11 [8.9, 14]	12 [9.7, 14]	11 [8.6, 15]	0.85
ALT, U/L	18 [13, 25]	18 [13, 28]	16 [12, 22]	20 [15, 25]	0.21
AST, U/L	21 [18, 27]	21 [18, 29]	21 [18, 25]	22 [20, 26]	0.65
TG, mmol/L	1.4 [0.88, 1.8]	1.6 [1.3, 1.9]	1.3 [0.81, 2]	1.3 [1, 1.7]	0.14
Creatinine, umol/L	61 [52, 75]	63 [52, 78]	60 [51, 71]	63 [54, 82]	0.38
BUN, mmol/L	5.8 [4.8, 7.3]	5.6 [4.8, 8.4]	5.7 [4.5, 6.1]	6 [5.2, 7.5]	0.12
Na+, mmol/L	142 [140, 143]	142 [140, 143]	142 [140, 143]	142 [140, 143]	0.88
Ca^2+^, mmol/L	2.3 [2.3, 2.4]	2.3 [2.2, 2.4]	2.3 [2.3, 2.4]	2.4 [2.3, 2.5]	0.19
D-Dimer, mg/L	0.46 [0.28, 0.82]	0.5 [0.28, 0.98]	0.43 [0.27, 0.75]	0.5 [0.3, 0.85]	0.53

a Compared to the deficiency group *p* < 0.05.

b Compared to the insufficient group *p* < 0.05.

mSBP, mean systolic blood pressure; mDBP, mean diastolic blood pressure; NBPDR, nocturnal blood pressure drop rate; BMD, bone mineral density; WBC, White blood cell; RBC, Red blood cell; IPTH, intact parathyroid hormone; PINP, Procollagen I N-Terminal Propeptide; ALT, alanine aminotransferase; AST, Aspartate aminotransferase; TG, triglyceride; TC, total cholesterol; LDL-C, low density lipoprotein cholesterol; HDL-C, high density lipoprotein cholesterol; BUN, blood urea nitrogen.

### 3.2 Association between 25-(OH) D3 and blood pressure

Because the data did not meet the homogeneity of variance, Spearman correlation test was used to calculate the correlation between 25-(OH) D3 and ambulatory blood pressure monitoring results, and the results are shown in Table 2. There is a negative correlation between 24 h mSBP, diurnal mSBP, nocturnal mSBP, maximum SBP and 25-(OH) D3. The Spearman partial correlation after CRP correction also had similar results.

**Table 2:** Spearman correlation test between 25-(OH) D3 and blood pressure.

	r_s_	*p*	r_s_^a^	*p*
24 h mSBP	−0.22	0.01	−0.21	0.02
24 h mDBP	−0.09	0.28	−0.08	0.36
Diurnal mSBP	−0.21	0.01	−0.19	0.02
Diurnal mDBP	−0.07	0.44	−0.06	0.49
Nocturnal mSBP	−0.21	0.01	−0.2	0.02
Nocturnal mDBP	−0.15	0.08	−0.14	0.10
NBPDR, SBP (%)	0.04	0.63	0.04	0.64
NBPDR, DBP (%)	0.12	0.21	0.1	0.25
Maximum SBP	−0.25	<0.01	−0.23	0.01
Maximum DBP	−0.1	0.24	−0.1	0.23
Minimum SBP	−0.1	0.23	−0.11	0.19
Minimum DBP	−0.04	0.66	−0.04	0.66

a Adjusted CRP.

### 3.3 Analysis of generalized additive model

**Figure 1:**
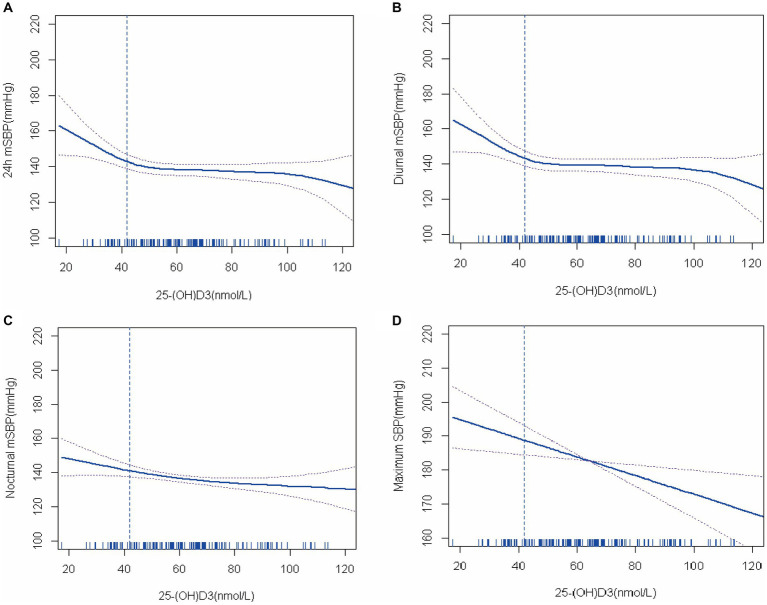
The relationship between 25-(OH) D3 levels and blood pressure (generalized additive model fitting linear regression).

The spearman correlation test found that 25-(OH) D3 was negatively correlated with ambulatory blood pressure, but the correlation was weak. Therefore, the generalized additive model (GAM) was further used to analyze whether there was a nonlinear relationship between 25-(OH) D3 and ambulatory blood pressure monitoring results. After using the GAM and fitting the smooth curve (after adjusting for CRP), we found that there was a nonlinear relationship between 25-(OH) D3 and 24 h mSBP, diurnal mSBP, nocturnal mSBP, respectively (Figure 1). However, maximum SBP and 25-(OH) D3 failed to establish a nonlinear relationship directly (likelihood test *p* = 0.06). For the model with nonlinear relationship, the data are fitted to the piecewise linear regression model to fit different slopes. We also use a typical linear regression model to fit the data and use the log likelihood ratio test to determine which model has the largest fitting. In this study, a recursive algorithm was used to determine the cutoff point of 25-(OH) D3 as 42 nmol/L, and then piecewise linear regression model was used to calculate the effect size (β) and 95% confidence intervals on both sides of the cutoff point. When 25-(OH) D3 ≤ 42 nmoL/L, 24 h mSBP decreased by 1.4 mmHg for every 1 unit increase in 25-(OH) D3 (β = −1.4, 95% CI: −1.7 ~ −1.1, *p* < 0.01, after adjusted CRP). However, when 25-(OH) D3 > 42 nmol/L, the relationship between 25-(OH) D3 and 24 h mSBP was not statistically significant. Similarly, non-linear relationships were found between 25-(OH) D3 and diurnal mSBP and nocturnal mSBP. On the left side of the cut-off point, for every 1 unit increase in 25-(OH) D3, the diurnal mSBP and nocturnal mSBP decreased by 1.4 mmHg (β = − 1.4, 95% CI: −1.7 ~ −1.2, *p* < 0.01, after adjusted CRP) and 1.1 mmHg (β = − 1.1, 95% CI: −1.5 ~ −0.82, *p* = 0.02, after adjusted CRP), respectively. Although the nonlinear model could not be established, there was still a certain correlation between 25-(OH) D3 and maximum SBP (β = −0.27 95% CI: −0.34 ~ −0.21 *p* = 0.01) (Table 3).

**Table 3:** The relationship between 25-(OH) D3 and blood pressure using a two-piecewise linear regression model.

	24 h mSBP			Diurnal mSBP			Nocturnal mSBP			Maximum SBP		
	β	*p*	*p*†	β	*p*	*p*†	β	*p*	*p*†	β	*p*	*p*†
Model	−0.2 [−0.3, −0.15]	<0.01		−0.2 [−0.3, −0.15]	<0.01		−0.19 [−0.24, −0.14]	0.01		−1.5 [−2.5, −0.44]	0.02	
Model*	−0.18 [−0.23, −0.13]	0.01		−0.18 [−0.22, −0.13]	0.01		−0.17 [−0.22, −0.12]	0.03		−0.27 [−0.34, −0.21]	0.01	
Model 1												
≤42 nmol/L	−1.4 [−1.7, −1.2]	<0.001	<0.01	−1.4 [−1.7, −1.1]	<0.01	<0.01	−1.2 [−1.5, −0.84]	0.02	<0.01	−1.5 [−2.5, −0.44]	0.02	0.06
>42 nmol/L	−0.08 [−0.13, −0.03]	0.30		0.08 [−0.13, −0.03]	0.30		−0.09 [−0.15, −0.03]	0.04		−0.17 [−0.35, 0.02]	0.14	
Model 1*												
≤42 nmol/L	−1.4 [−1.7, −1.2]	<0.01	<0.01	−1.4 [−1.7, −1.1]	<0.01	<0.01	−1.1 [−1.5, −0.82]	0.02	0.04	−1.5 [−2.7, −0.22]	0.02	0.06
>42 nmol/L	−0.06 [−0.11, −0.01]	0.45		−0.06 [−0.11, −0.01]	0.43		−0.08 [−0.13, −0.02]	0.39		0.16 [−0.39, 0.07]	0.17	

Model: Fitting model by standard linear regression; Model 1: Fitting model by two-piecewise linear regression; *Adjusted CRP; †: log-likelihood ratio test.

## 4 Discussion

Hypertension and osteoporosis are common chronic diseases in the aging people. At present, more and more studies have proved that there is an association between osteoporosis and hypertension (15, 16). Vitamin D plays an important role in calcium and phosphorus metabolism, which affects the occurrence of osteoporosis. Therefore, vitamin D deficiency can lead to osteoporosis. In addition, vitamin D deficiency has been found to be associated with hypertension. In this retrospective study, we sought to find the effect of vitamin D deficiency on blood pressure in elderly patients with both diseases. We enrolled 139 elderly hypertensive patients with osteoporosis and found that vitamin D deficiency was associated with higher 24 h mSBP, nocturnal mSBP, NBPDR DBP and maximum SBP. We first used spearman to analyze the correlation between blood pressure and vitamin D, and although the correlation between the two was confirmed, the correlation was weak. This suggests that there may be a nonlinear relationship between blood pressure and vitamin D. The generalized additive models were further used to investigated the relationship between Vitamin D and blood pressure and found a significant negative correlation between Vitamin D and 24 h mSBP, diurnal mSBP, nocturnal mSBP, maximum SBP. This suggests that vitamin D deficiency affects systolic blood pressure in such patients. To this end, we found that 42 nmol/L was the best cut-off point for Vitamin D level. On the left side of the cut-off point, 24 h mSBP, nocturnal mSBP and diurnal mSBP increased significantly as Vitamin D level decreased. This suggests that vitamin D supplementation may be beneficial in lowering blood pressure, especially systolic blood pressure, in elderly patients with osteoporosis when vitamin D levels are below 42 nmol/L.

Vitamin D is associated with cardiovascular health, including hypertension (17–19). Several studies have shown a correlation between vitamin D and hypertension. In the Third US National Health and Nutrition Examination Survey, there was a significant negative correlation between vitamin D and blood pressure, especially in older adults (20). Forman et al. found that plasma vitamin D levels were inversely and independently associated with the risk of developing hypertension (21). In a prospective study, Goel et al. demonstrated that vitamin D supplementation has a blood pressure-lowering effect in hypertensive patients and should be supplemented concomitantly with anti-hypertensive drugs. Sheikh et al. randomized 171 patients with hypertension to vitamin D supplementation or placebo and showed a significant blood-pressure-lowering effect of vitamin D supplementation in patients with hypertension and vitamin D deficiency (22). In another study, Dalbeni et al. investigated the effect of vitamin D supplementation in patients with heart failure and vitamin D deficiency and found that systolic blood pressure was significantly lower in those who received 4,000 units of vitamin D daily for 6 months compared with placebo group (23).

At present, the specific mechanism of vitamin D affecting blood pressure has not been fully elucidated, and there are several potential mechanisms. First, vitamin D may regulate blood pressure through the RAAS system. In animal experiments, lack of vitamin D receptor can increase renin and angiotensin II in mice (24). Second, vitamin D deficiency can cause hyperparathyroidism, and elevated parathormone can lead to elevated blood pressure (25). Third, vitamin D deficiency is associated with insulin resistance, which is thought to be associated with the development of hypertension (26, 27). In addition, it has been suggested that vitamin D deficiency may affect blood pressure by impairing endothelial cell function (28). On the contrary, it has also been suggested that vitamin D may be used as a marker of sunlight to reflect the effect of sunlight on blood pressure because sunlight can release NO stored in the human skin and affect blood pressure (18, 29).

Osteoporosis is also a common disease in the elderly. Similarly, vitamin D deficiency plays an important role in osteoporosis (30). Vitamin D is related to calcium and phosphorus metabolism. Vitamin D supplementation is an important means to treat osteoporosis and prevent fractures in patients with osteoporosis (31, 32).

Hypertension and osteoporosis are common diseases in the elderly, and have been confirmed to be related to vitamin D. The relationship between vitamin D and blood pressure in hypertensive patients with osteoporosis has not been studied yet. We explored this and found that vitamin D deficiency showed a non-linear and negative relationship with blood pressure in such patients, especially systolic blood pressure. When below 42 mmol/L, blood pressure increased significantly with the decrease of vitamin D, while above 42 mmol/L, there was no significant change. This suggests that vitamin D supplementation and regular testing are warranted in such patients.

## 5 Limitation

There were a few limitations and potential biases in this trial as follows. First patients were enrolled in the study from a single-center study. This issue may raise the possibility of selection bias. Second, this is a retrospective study, although the relationship between vitamin D and blood pressure in osteoporosis patients has been found, the effect of vitamin D supplementation on blood pressure in such patients has not been further studied, and further prospective studies are needed to confirm this. Third, because of the differences in vitamin D levels among different populations, in countries such as India, Pakistan, and Afghanistan, the majority of the population has Fitzpatrick skin type V or VI, which leads to less efficient conversion of vitamin D from ultraviolet radiation, resulting in lower vitamin D levels than light-skinned people (33). The effect of vitamin D on blood pressure needs to be further confirmed by large multi-ethnic studies in the future.

## Data availability statement

The raw data supporting the conclusions of this article will be made available by the authors, without undue reservation.

## Ethics statement

The studies involving humans were approved by the Ethics Committee of the Zhongshan Hospital Affiliated to Xiamen University. The studies were conducted in accordance with the local legislation and institutional requirements. The Ethics Committee/Institutional Review Board waived the requirement of written informed consent for participation from the participants or the participants’ legal guardians/next of kin because the study design did little harm to the subjects and the retrospective study was difficult to obtain written informed consent.

## Author contributions

TW: Writing – original draft, Methodology, Investigation, Formal analysis, Data curation. ZL: Writing – review & editing, Methodology, Investigation, Formal analysis, Data curation, Conceptualization. CW: Writing – review & editing, Formal analysis, Resources. XL: Writing – review & editing, Methodology, Formal analysis, Data curation.
